# Homœopathy Triumphant

**Published:** 1889-12

**Authors:** L. L. Helt


### HOMŒOPATHY TRIUMPHANT.

When the Hon. J. B. Foraker became Governor of Ohio he appointed a homœopathic medical staff at the Penitentiary. As they are about to give place to the appointees of the Governor-elect, the present is a favorable time to compare results between their management and that of their predecessors of the allopathic faith. If the following is an indication of the superiority of one school over another, homœopathists have reason to be proud of their brethren of the Penitentiary.

In 1885 Dr. C. R. Montgomery, allopath, was in charge. His mortuary list for that year contains thirty-nine names. Seven months of 1886, under the same *regime*, has a death list of eighteen. The remaining five months of that year Dr. Clemmer, homœopath, was in charge, and but three were added to the gross mortality. In 1887, 1888, and 1889 there were eighteen, nineteen, and twenty deaths respectively—each of these years showing an increase in the prison population over the preceding year.

In his annual report for the year 1889 to the Board of Managers, Dr. Clemmer writes :

" From the mortuary list it is observed that there were twenty deaths, three from suicide, one by violence, and sixteen from natural causes. This is a low rate of mortality considering the character of the subjects, but it is made higher through an increased ratio of sickness and death among the United States' prisoners as compared with the State prisoners. The Federal prisoners, for the most part, come from warmer climates of the South and Southwest. It is noticeable that these men, including the Indian population, are prone to incur diseases of the respiratory organ. A want of acclimation coupled to the unfavorable conditions of prison life have caused an undue amount of sickness and death among United States prisoners than among the State prisoners. The gross population of State prisoners for the year is two thousand and forty-two with nine deaths from disease, or 0.40 of 1 per cent. The gross population of United States prisoners is two hundred and fifty-three with seven deaths, or 2.77 per cent.

"The death-rate for the year is $8\frac{7}{10}$ in the thousand."

This low death-rate is remarkable, particularly when it is a fact that criminals are far below the general average in physique and that they are rarely free from constitutional affections of a poisonous type. Their environments, more especially in old institutions like the Ohio Penitentiary, are not conducive to the maintenance of good health; on the contrary, they are apt to foster and produce certain classes of disease.

A comparison of the death-rates of twenty-five penal institutions in different parts of the Union to impeach the claim advanced in the daily press of the State that the sanitary condition of the Penitentiary was so bad as to render it unfit for the habitation of even animals, revealed the fact that Ohio's was the least. This is another substantial testimony to the superiority of Homœopathy. Another very strong argument in favor of our school is the fact that the expense of running the medical department averaged thirteen hundred dollars a year less than when it was under allopathic control.

Orificial surgery was introduced in the treatment of chronic affections. Sixty-seven cases were operated upon, satisfactory results following in all but three cases, and these failures Dr. Clemmer attributes to his own lack of skill rather than to any defect in the system.

Dr. Clemmer is a graduate of Pulte College, Cincinnati, and besides the subscriber, who is a graduate of the same college, he has the assistance of Dr. Howell, a graduate of the Cleveland College.

L. L. Helt, M. D.

